# Potential use of other starch sources in the treatment of glycogen storage disease type Ia – an in vitro study

**DOI:** 10.1186/s13023-024-03201-1

**Published:** 2024-07-30

**Authors:** Vaneisse Monteiro, Karina Colonetti, Carlos Henrique Pagno, Helena OS Schmidt, Fernanda Sperb-Ludwig, Bibiana Mello de Oliveira, Soraia Poloni, Alessandro O Rios, Carolina F Moura de Souza, Ida Vanessa Doederlein Schwartz

**Affiliations:** 1 Postgraduate Program in Genetics and Molecular Biology, Universidade Federal do Rio Grande do Sul, Porto Alegre, Brazil; 2 Basic Research and Advanced Investigations in Neurosciences Laboratory (B.R.A.I.N), Hospital de Clínicas de Porto Alegre, Porto Alegre, Rio Grande do Sul Brazil; 3 Bioactive Compounds Laboratory, Institute of Food Science and Technology, Universidade Federal do Rio Grande do Sul, Porto Alegre, Brazil; 4 Medical Genetics and Nutrition Services, Hospital de Clínicas de Porto Alegre, Porto Alegre, Rio Grande do Sul Brazil; 5 Department of Genetics, Universidade Federal do Rio Grande do Sul, Porto Alegre, Brazil; 6 Center for Clinical Research, Nuclimed, Hospital de Clínicas de Porto Alegre, Porto Alegre, Rio Grande do Sul Brazil; 7 Undergraduate program in Food Technology, Universidade Federal de Ciências da Saúde de Porto Alegre, Porto Alegre, Rio Grande do Sul Brazil; 8 Serviço de Genética Médica, Hospital de Clínicas de Porto Alegre, Rua Ramiro Barcelos, Porto Alegre, 2350, 90035-003 RS Brazil

**Keywords:** Hepatic glycogen storage disease, Therapeutic strategies, Cornstarch, Sweet manioc starch, Sugar, Amylopectin

## Abstract

**Background:**

Glycogen storage disease type Ia (GSD-Ia) is one of the most common hepatic GSD. Its treatment mainly consists of a diet including a high intake of slow-digestion carbohydrates such as raw cornstarch and the restriction of simple sugars. This enables the maintenance of euglycemia and prevents secondary metabolic disorders. Starch is a glucose polymer formed by amylose and amylopectin, which can be obtained from distinct sources. Although uncooked cornstarch has been successfully used in the treatment of GSD-Ia, it can lead to hyperglycemia and weight gain. in vitro andin vivo tests indicated that sweet manioc starch can be potentially used in the treatment of GSD-Ia.

**Results:**

The moisture analysis revealed a variation from 10.3 to 12.8% in the sweet manioc starch samples, whereas the moisture content of uncooked cornstarch ranged from 7.3 to 11.1%. Quantifiable sugar was detected in 3/5 samples of sweet manioc starch and 1/3 samples of uncooked cornstarch. Notably, this uncooked cornstarch brand is widely employed in GSD-Ia treatment in Brazil. Products B and E had higher values of amylopectin and undetectable levels of sugars. A clinical trial is warranted to compare samples F and G and determine the impact of sugar trace in the same dietary source of starch.

**Conclusions:**

Collectively, the results demonstrated possible therapeutic alternatives for GSD-Ia in addition to traditional uncooked cornstarch.

**Supplementary Information:**

The online version contains supplementary material available at 10.1186/s13023-024-03201-1.

## Background

Hepatic glycogen storage diseases (GSD) are a group of genetic diseases in which glycogen degradation is hampered [1]. GSD type Ia (GSD-Ia) is one of the most common types, caused by the deficient activity of the enzyme glucose-6-phosphatase (OMIM #232200) [1–4]. The impairment of the gluconeogenesis pathway in GSD-Ia results in an inability to maintain glucose homeostasis during fasting periods or in between meals, leading to potentially life-threatening hypoglycemia. Hypoglycemia in GSD-Ia can give rise to a spectrum of symptoms, including fatigue, seizures, and, if left unmanaged, the potential for life-threatening consequences. Moreover, this disease may present additional symptoms and complications. Renal injury, characterized by proteinuria and hypertension, is observed, as is bone disease, which may result in growth retardation, and adenomas [5, 6].

**Fig. 1:**
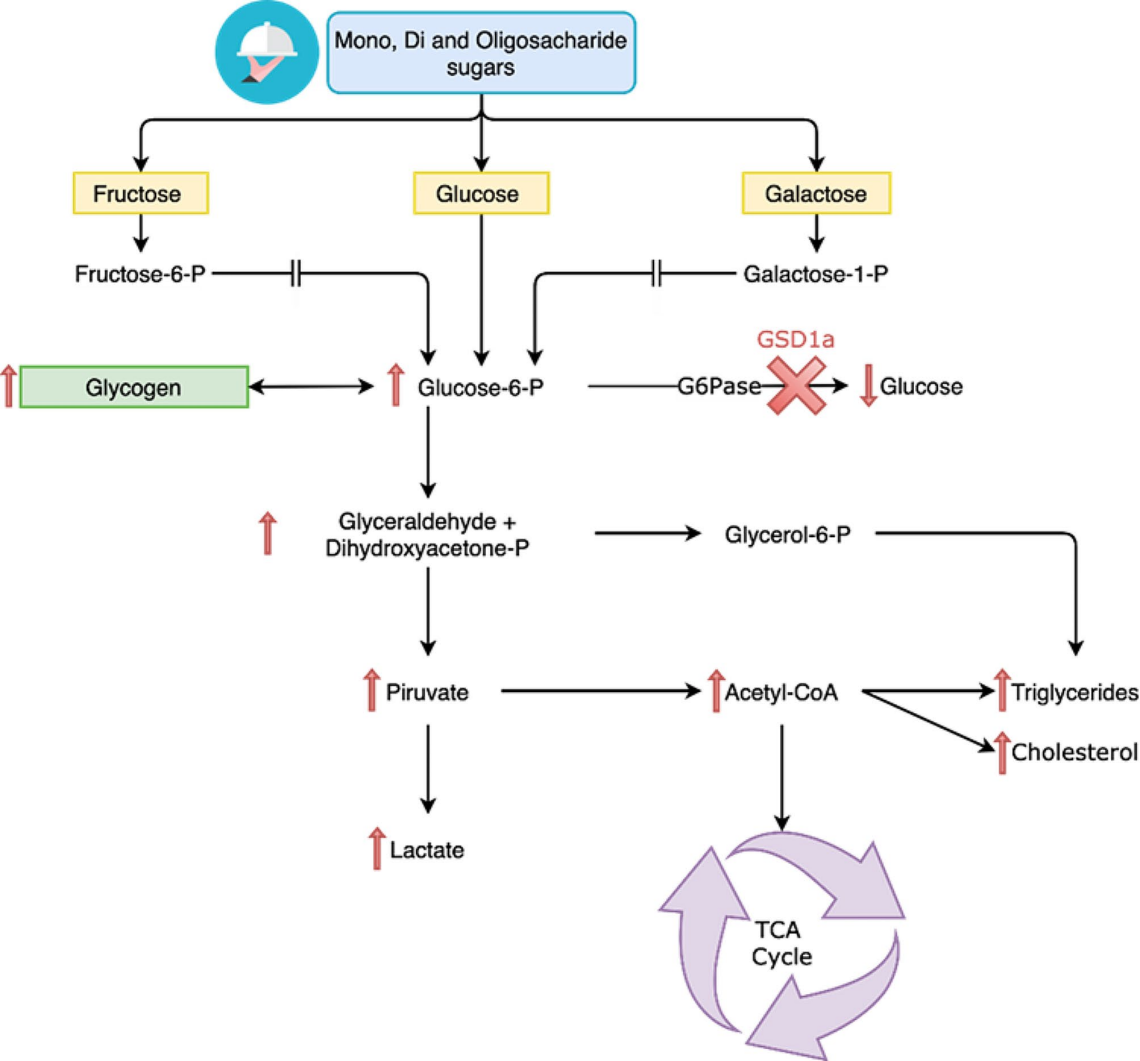
Schematic metabolism of monosaccharides metabolism and the impact on GSD-Ia. After carbohydrate ingestion, the monosaccharides (D-glucose, D-fructose and D-galactose) lead to the formation of D-glucose-6-phosphate. Due to the deficient activity of the glucose-6-phosphatase enzyme, the D-glucose remains in the intracellular fluid, thus stimulating the glycogen formation, ultimately causing hypoglycemia and an excessive production of lactate. GSD1a: glycogen storage disease type 1a; P: phosphate; TCA: tricarboxylic acid cycle

The treatment of GSD-Ia is mainly based on dietary management and aims to maintain normoglycemia while preventing secondary metabolic disorders [1, 5, 6]. Sugars that are rapidly hydrolyzed (e.g., sucrose, fructose, and lactose) are restricted according to international guidelines once they increase the hepatic glycogen storage [1, 5, 6]. Since the 1950s, a diet rich in monosaccharides and disaccharides has been known to lead to increased lactate levels in individuals with GSD-Ia [7, 8] (Fig. 1). However, no consensus yet exists on the maximum usable amount of these sugars in the treatment [6, 9, 10].

Administration of uncooked cornstarch (UCCS) is the main current therapeutic strategy for GSD-Ia due to its slow digestion and consequent efficiency in maintaining euglycemia [6, 11]. Starch is a glucose polymeric molecule (polysaccharide), whose enzymatic breakdown leads to a slow release of mono-, di-, and oligosaccharides [12].

Commercially available starch samples are extracted from various sources, mostly corn and cassava. Sweet manioc starch (SMS), also known as tapioca gum [13, 14] or cassava starch, is extracted from unfermented cassava roots following a particular technological process [15]. The pure starch is a mixture of amylose and amylopectin, thus impacting its digestibility [16, 17].

Although the use of UCCS in the treatment of GSD-Ia has been successful, a UCCS-induced high glycemic index has been reported in these patients [10, 18, 19]. The therapeutic rationale for using UCCS is to provide the maintenance of euglycemia throughout the night, be palatable and easy to manipulate, lead to limited adverse effects (especially regarding weight gain), and not interfere with the appetite [19]. However, from a clinical perspective, the adverse events of the high intake of UCCS include hyperinsulinemia and obesity [20].

The efficacy of uncooked starch samples from distinct sources in maintaining nocturnal euglycemia has previously been evaluated in patients with GSD-Ia. Of all the evaluated samples (50% amylose-containing cornstarch, sweet manioc, potato, rice, wheat, and arrowroot starches), UCCS was the most effective in maintaining normoglycemia in patients with GSD-Ia [21]. The authors also demonstrated that not all starch samples were properly hydrolyzed by the patients.

Interestingly, the search for alternative therapeutic dietary strategies must not only meet social demands but also include products that are affordable and regionally available and could be used by patients with some degree of intolerance to the established treatment [22, 23].

Recently, the therapeutic potential of SMS has been suggested by an in vitro dynamic small-intestine model (TIM-1) [24]. These preclinical results encouraged assessment of the safety and efficacy of SMS in patients with GSD-Ia. In a triple-blinded, randomized clinical trial, SMS maintained euglycemia for longer periods compared to UCCS. However, an increase in lactate levels occurred even in the absence of hypoglycemia and regardless of the starch sample. The authors hypothesized that such increased lactate concentrations could be directly associated with the metabolism of fructose or other sugars that could be present in the studied starch, indicating the need for further studies [25].

Within this context, this study aimed to biochemically characterize four brands of SMS (A, B, C, and D), a lyophilized tapioca gum (E), two brands of cornstarch (UCCS) made in Brazil (F and G), and one brand of UCCS made in the United States (H) regarding the amounts of moisture, sugar (sucrose, fructose, and glucose), amylose, amylopectin, lipids, and proteins.

## Methods

**Fig. 2:**
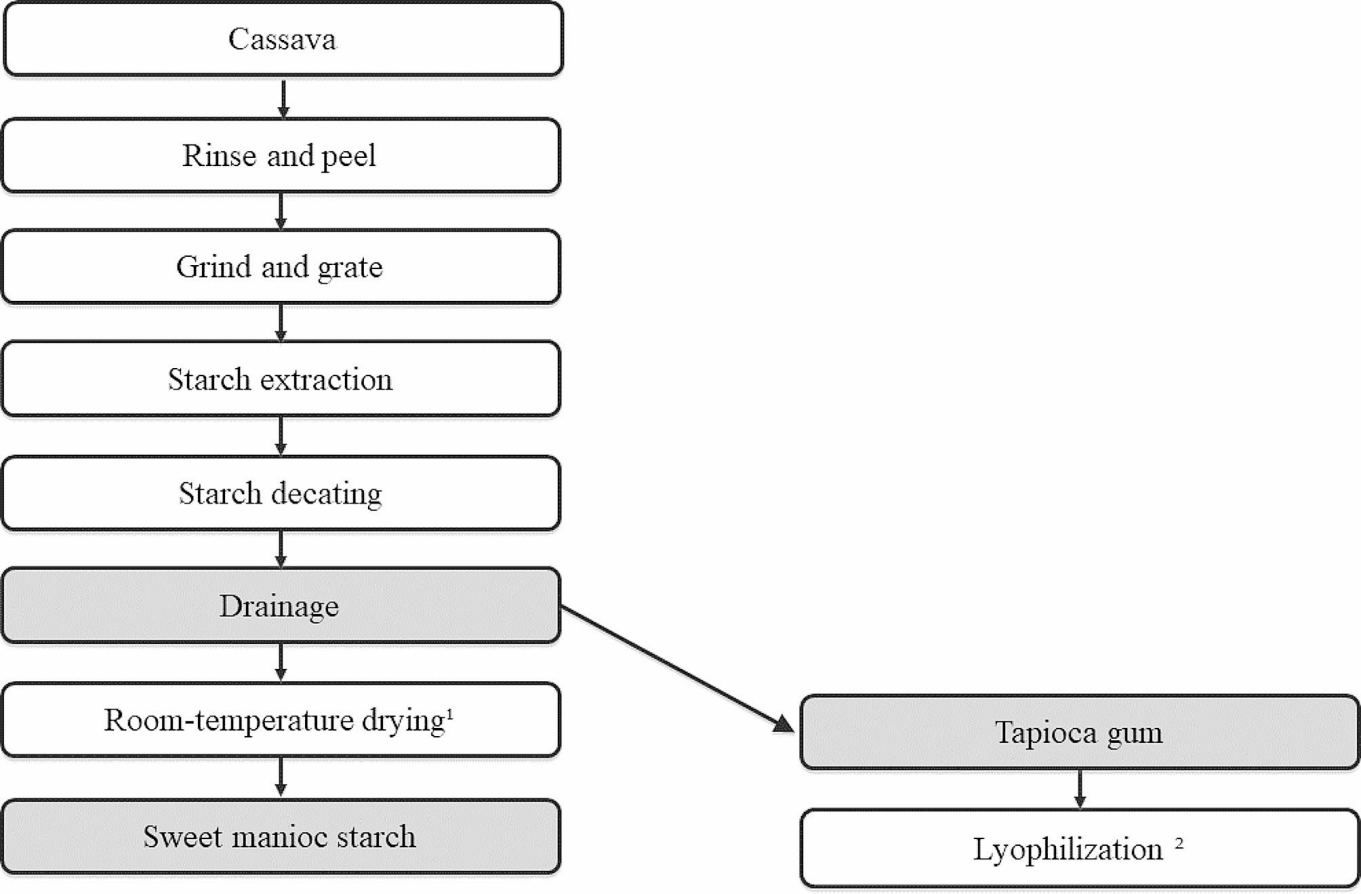
Production flow of the sweet manioc starch and tapioca gum: conventional drying process¹ and employed method for sample conservation²

Samples A, B, C, and D were obtained in supermarkets in Rio Grande do Sul – the Brazilian southernmost state. Sample E was obtained in Pará (North Brazil), while samples G and H were obtained in São Paulo (Southeast Brazil) and in the United States, respectively. After the purchase, the products were kept in a cool, dry place and protected from light until analysis. For product E, lyophilization was conducted (Liobras, L101, Liotop, São Carlos, SP, Brazil) to increase conservation (Fig. 2). All tests were performed using sample triplicates, except for the amylose/amylopectin rate, which was analyzed as sample duplicates [26–30]. Results are presented as value mean ± SD.

### Extraction and quantification of sugars

Monosaccharides (D-fructose and D-glucose) and sucrose were quantified using high-performance liquid chromatography (Waters Alliance 2695®, Milford, USA) connected to a refractive index detector (2414, with Aminex® HPX-87 H column, 300 mm x 7.8 mm, Bio-Rad Laboratories Inc, Hercules, California, USA). The mobile phase used was H2SO4 0.005 M. The chromatographic conditions were those described by Petkovsek et al. [31] with adaptations (Supplementary material).

### Amylose and amylopectin content

Total starch and amylose content were obtained by ConA precipitation procedure, using the commercial amylose/amylopectin assay kit (Megazyme Co., Wicklow, Ireland). Absorbance was read at 510 nm, according to the manufacturer’s instructions. Amylopectin content was obtained by the difference between total starch and amylose content. All values were expressed in percentage (%).

### Moisture, protein, and lipid content

The contents of protein, ether-extractable lipids, and moisture were determined using the standard Association of Official Analytical Chemists methods [32]. The total protein content was determined by the Kjeldahl method using a correction factor of 6.25. The lipid content was determined using a Soxhlet extractor (Foss Soxtec, model 2055, Denmark). The moisture was determined by the gravimetric method, by drying the samples in an oven (DeLeo oven, model TLK 48, Porto Alegre, Brazil) at 105 ºC until constant weight. Previously dried metal capsules were used [31, 32,33]. The sample characteristics, batch numbers, and expiration date are described in Supplementary Table 1.

## Results

The chemical composition of the analyzed samples is detailed in Table 1.

**Table 1:** – Biochemical characterization of the starch samples

	Samples	Batch number	Moisture (%)	Amylose (%)	Amylopectin (%)	Amylose/ amylopectin	Sugar (g/100 g)*	Glucose (g/100 g)²	Fructose (g/100 g)³	Saccharose (g/100g)^4^
SMS	A^a^	001–18¹	10.9 ± 0.11	19.2 ± 0.74	80.8	0.24	40 ± 0.01	15 ± 0.00	25 ± 0.01	-
	A^b^	001–18¹	10.5 ± 0.09	21.1 ± 2.02	78.9	0.27	38 ± 0.00	14 ± 0.00	24 ± 0.00	-
	A^c^	001–18¹	11.5 ± 0.06	20 ± 0.98	80.0	0.25	30 ± 0.14	15 ± 0.01	23 ± 0.00	-
	B	5,923,346	10.8 ± 0.13	18.8 ± 0.18	81.2	0.23	-	-	-	-
	C	004	11.9 ± 0.17	18.1 ± 0.31	81.9	0.22	28 ± 0.07	13^†^	24 ± 0.00	-
	D	C19BRVP263	10.3 ± 0.26	20 ± 1.82	80	0.25	39 ± 0.00	15 ± 0.00	24 ± 0.00	-
	E	19.GMA.049**	12.8 ± 0.36	19.2 ± 1.07	80.8	0.24	-	-	-	-
UCCS	F	63 C, 67 C	11.1 ± 0.31	22.1 ± 1.23	77.9	0.28	22 ± 0.14	-	14 ± 0.00	24^†^
	G***	2,607,202,019	10.1 ± 0.34	23.1 ± 2.27	76.9	0.30	-	-	-	-
	H	174D9	7.3 ± 0.21	23.4 ± 2.4	76.6	0.31	-	-	-	-

SMS: Sweet manioc starch; UCCS: Uncooked cornstarch

¹There was a unique annual batch number for the sample A (sweet manioc starch Fritz e Frida® although the manufacture and the expiration dates are different among the samples, as indicated by ^a^, ^b^ and ^c^

*Total amount of sugar in the dried material

**Tapioca gum: drained sweet manioc starch before the room-temperature dry process. For this study, we conducted a lyophilization to avoid water excess

***French brand made in Brazil

^†^Detected in only one triplicate test

Detection range (DR) and quantification range (QR). ²Saccharose: DR = 0.032 g/100 g and QR = 0.097 g/100 g; ³glucose DR = 0.022 g/100 g and QRv = 0.067 g/100 g; [4]fructose DR = 0.010 g/100 g and QR = 0.029 g/100 g.

(-): values below detection range

The SMS samples had an average moisture content between 10.3% and 12.8% and sugar content below the detection limits in two tested brands (B and E). For the remaining brands, the average sugar content was higher than that found in UCCS (28–40 g/100 g vs. 22 g/100 g, respectively). The amylopectin content ranged from 78.9 to 81.9%.

For UCCS, the moisture was 10.1% and 11.1% in samples F and G and 7.3% in sample H. The presence of sugars was detected only in sample F. The highest value of amylopectin was found in sample F (Table 1).

The carbohydrate content declared on the product label by the manufacturers in the samples was E = 90% (the original product, not lyophilized), H = 87.5%, C = 86%, in samples A, B, and F = 85%, and D = 80%. Regarding sample G there were no nutrition facts on the packaging.

The analysis of the protein and lipid content indicated that the starch samples only presented traces of these chemical compounds, regardless of the source.

## Discussion

To the best of our knowledge, this is the first study evaluating the sugar content of starch samples extracted from distinct sources used in the treatment of GSD-Ia. The feasibility of a new therapeutic strategy for this disease, obtained by the lyophilization of the tapioca gum (sample E), was also examined. The study additionally biochemically characterized the samples for moisture, amylose, and amylopectin content, as well as proteins and lipids.

In GSD-Ia, the mechanism that regulates the synthesis and degradation of glycogen is impaired, leading to the accumulation of this polymer in the liver [6]. Experts recommend small, frequent meals, rich in complex carbohydrates, and the restricted ingestion of monosaccharides [6].

Despite the lack of consensus on the maximum usable amount of sugar in treatment, it is known to health professionals involved in the care of patients with GSD type I that sucrose, fructose, and galactose in the diet result in high concentrations of lactate and acidosis [6, 9, 10].

Periodic administration of UCCS is also recommended, and the dosage is calculated in accordance with the body mass and age of the patient [5, 6]. In a recent study, our group reported that the mean consumption of UCCS for male patients was 77.5 g/dose (465 g/day)^25^.

While UCCS has demonstrated efficacy in the management of GSD-Ia, documented instances indicate an association with a heightened glycemic index in affected individuals [10, 18, 19]. This observation prompts consideration of potential risks, including hyperglycemic spikes, occasionally followed by rebound hypoglycemia. It is noteworthy that sustained hyperglycemia may result in elevated insulin levels, raising concerns regarding the stability of blood glucose levels. This instability is characterized by pronounced spikes in blood sugar, predisposing patients to potential episodes of hypoglycemia [10, 18, 19].

Lyophilized tapioca gum (sample E) presented the highest moisture. This may result from its manufacturing processes, which consist of draining the cassava mass before the drying step (Fig. 2). In this study, the lyophilization of the tapioca gum was conducted at low pressure and temperature to avoid oxidation and enzymatic reactions. This process assists in food conservation without losing its characteristics due to heating, such as gelatinization that increases its availability to amylase, affecting digestibility [17, 26]. The original product, not lyophilized, likely has a higher moisture content than that described.

Cassava starch contains nearly 80% amylopectin and 17–20% amylose. It also contains approximately 170 g/kg of sucrose and traces of fructose and dextrose [27]. This study corroborated previous data regarding the amylose and amylopectin content and added the new information that a high amount of fructose and glucose (but not sucrose) were found. However, the content of these reducing sugars may be influenced by the cassava variety, the time of the harvest, and the post-harvest storage period. Higher sugar amounts have been related to the low amounts of starch and high water content in the roots [28, 29].

A previous study using the TIM-1 model indicated the therapeutic potential of SMS since it demonstrated a lower amount of rapidly available glucose followed by a higher content of resistant starch compared to UCCS, thus resulting in limited glucose release and a greater amount of undigested material [24]. The ratio between amylose and amylopectin was not sufficient to explain the difference found in the digestion pattern of this product in the evaluated model [23]. Based on studies conducted by Nalin et al. [23, 24], two brands of starch were selected to be tested in the clinical trial: A (SMS) and F (UCCS) [25].

The amylose-to-amylopectin ratio, along with the distribution and chain length of amylopectin, constitutes pivotal factors influencing the digestibility of starches. This is particularly pertinent not only in the broader context of starch digestion physiology but also in therapeutic interventions such as the management of hepatic GSD [23, 24].

The study by Nalin et al. [24] could not evaluate the lactate levels in the TIM-1 model. The blood lactate levels after the consumption of UCCS and SMS were investigated by our group in the triple-blinded trial [25]. Both UCCS and SMS induced an increase in lactate levels in the absence of hypoglycemia. The lowest mean lactate levels throughout the trial occurred using UCCS but did not reach statistical significance.

The amylopectin levels in samples A and F (used in the clinical trial [25]) were similar. Even though reducing sugar amounts were detected in both samples, sample F seemed to have more, potentially contributing to a putative increase in blood lactate levels.

The starches evaluated can also be characterized according to their source. UCCS is derived from grain, whereas SMS is derived from tubers. Both have a circular granule shape, but UCCS is polygonal and SMS is irregular. The amylose content varies from 25 to 28% in UCCS and 17–20% in SMS. The gelatinization temperature in UCCS ranges from around 62 °C to 80 °C, whereas in SMS it is lower, around 52 °C to 65 °C. These factors influence the digestibility and metabolism of starch [27, 34].

Our investigation revealed that in SMS samples B and E, the sugar content was below detectable limits. This is especially crucial for GSD1 patients who must regulate their sugar intake due to their impaired ability to break down glycogen effectively [5, 6]. Although the remaining SMS brands showed slightly higher sugar content compared to UCCS, these levels remain relatively low, suggesting that SMS sources could be suitable for GSD patients in need of minimizing sugar intake. Both SMS and UCCS samples exhibited a notably high amylopectin content. Amylopectin, being a branched form of starch, serves as a gradual source of glucose when required by the body [23, 24]. This high amylopectin content is advantageous for GSD patients, providing a slow and steady release of glucose, that aligns well with their needs which could provide an extension of the interval between feedings, minimizing the impact on the quality of life of patients who require a frequent exogenous source of glucose to reduce the risk of hypoglycemia and consequent risk of death [10, 19–21, 25]. The combination of low or no sugar content and high amylopectin content, in these starch sources makes them a promising choice for GSD patients.

Our data suggest that investigating the therapeutic use of SMS using samples B or E would be more appropriate than the other samples because these displayed higher amylopectin levels and non-complex sugars were not quantifiable. Another possibility would be a clinical trial comparing the use of sample F (widely prescribed for the treatment of GSD-Ia in Brazil) vs. sample G to examine the influence of the sugar content on blood lactate levels and glycemic control after the intake of UCCS. Collectively, our data demonstrate possible therapeutic alternatives for hepatic GSD-Ia, in addition to traditional UCCS, and reinforce the need for new clinical trials.

### Electronic supplementary material

Below is the link to the electronic supplementary material.

Supplementary Material 1

## Data Availability

The manuscript has data included as electronic supplemental material and additional data will be available on reasonable request.

## Declarations

*Human and Animal Rights and Informed Consent*.

This article does not contain any studies with human or animal subjects performed by any of the authors.

## Abbreviations

GSD: Glycogen storage disease
GSD-Ia: Glycogen storage disease type Ia
UCCS: uncooked cornstarch
SMS: sweet manioc starch
TIM-1: dynamic small intestine model
HPLC: high performance liquid chromatography
DR: Detection ranges
QR: quantification ranges
AOAC: Association of Official Analytical Chemists
