# Supplementary material for: Potential use of other starch sources in the treatment of glycogen storage disease type Ia – an in vitro study
Source: Orphanet J Rare Dis. 2024 Jul 30;19:283. doi: 10.1186/s13023-024-03201-1 (PMC11289971; doi:10.1186/s13023-024-03201-1)
Supplement: Supplementary file 1 — Supplementary Material 1 [file 13023_2024_3201_MOESM1_ESM.docx]

**Supplementary table** – Commercial data of the analyzed samples of starches

| Sample |  | Brand | Batch number | Expiration date |
| --- | --- | --- | --- | --- |
| Sweet manioc starch | A^a^ | Fritz e Frida^®^ | 001-18 | August, 2020 |
|  | A^b^ | Fritz e Frida^®^ | 001-18 | May, 2020 |
|  | A^c^ | Fritz e Frida^®^ | 001-18 | January, 2021 |
|  | B | KiSabor^®^ | 5923346 | August, 2020 |
|  | C | Caldo Bom^®^ | 004 | May, 2021 |
|  | D¹ | Yoki^®^ | C19BRVP263 | March, 2020 |
|  | D² | Yoki^®^ | E20BRVP151 | May, 2021 |
|  | E | Regional tapioca gum^®^ (lyophilized^d^) | 19.GMA.049 | - |
|  |  |  |  |  |
| Uncooked corn starch | F¹ | Maizena^®^ | 63C | December, 2021 |
|  | F² | Maizena^®^ | 67C | February, 2021 |
|  | G | Tereos^®^ | 2607202019 | July, 2019 |
|  | H | Argo^®^* | 174D9 | July, 2022 |

The manufacture and the expiration dates are different among the samples, as indicated by ^a^, ^b^ and ^c^. E: Handmade sample submitted to lyophilization before drainage. *Imported sample (from the United States).
